# The Level of Evidence in Two Leading Endodontic Journals

**Published:** 2013-01-20

**Authors:** Leila Shafiei, Arash Shahravan

**Affiliations:** 1 Oral and Dental Diseases Research Center, Kerman University of Medical Sciences, Kerman, Iran

**Keywords:** Clinical Trials, Dentistry, Endodontics, Evidence Based, Journal Article

## Abstract

**Introduction:**

The successful practice of dentistry, including endodontics, relies on a wide spectrum of dental research. The quantity and quality of research evidence in endodontics have seldom been evaluated. The aim of this study was to evaluate the level of evidence in current leading endodontic journals.

**Materials and Methods:**

All the articles published in 2000, 2006 and 2010 in two major endodontic journals (Journal of Endodontics and International Endodontic Journal) were evaluated. These articles were classified according to the level of evidence (LOE) using Oxford Scale from 0 to 5 and type of the study.

**Results:**

Of the articles assessed, 3.2% were clinical trials, 47.8% were experimental, 5.6% were animal studies and 43.4% were of other types. Subdivisions according to LOE were 4.3% as level 1, 0.9 % level 2, 7.3% level 3, 0.4% level 4 and 3.5% level 5. Overall, 83.6% of the articles were classified as “non-evidence-based”. There was a marginally significant increase in the percentage of articles with high level of evidence in recent years.

**Conclusion:**

There is a substantial shortage of articles with high level of evidence in clinical endodontics. However, there was a gradual increase in the number of high LOE articles published in both journals.

## 1. Introduction

Evidence-based medicine was defined by Sackett in 1996 [1]. It is explained as the application of best available scientific evidence to select the most appropriate treatment for an individual patient [2]. The emphasis is on the necessity to increase the individual physician’s clinical experience with valid external evidence originating from articles with high level of evidence such as randomized clinical trials (RCT) [3]. The American Dental Association defined evidence-based dentistry as “an approach to oral healthcare that requires the judicious integration of systematic assessments of clinically relevant scientific evidence, relating to the patient’s oral and medical condition and history, with the dentist’s clinical expertise and the patient’s treatment needs and preferences” [4]. In a recent review of practical applications and implications of evidence-based dental practice, it was stated that “evidence-based dentistry involves preparing a systematic review of relevant, reliable research studies on the treatment being assessed” [5]. By using evidence-based dentistry, clinicians can ultimately determine the best treatment plan based on the best available evidence.

Fletcher [6, 7] for the first time described how evidence could be ranked , into different levels and grades to give an idea of the quality of the evidence in terms of minimizing bias and flaws. These levels have been developed over the ensuing years. The ranking system, based on the level of evidence, can be used to rate a study according to the quality of scientific evidence presented in the article. The National Health Service Research and Development Center for Evidence-based Medicine (RDC) in Oxford, UK, developed an updated version [6, 7].

Lau and Samman assessed the relationship between LOE and journal impact factor (IF) in oral and maxillofacial surgery (OMFS) journals [6]. They reported a statistically significant correlation between LOE and IF. Among the five LOE groups the majority (50%) were categorized as non-evidence, followed by case series (40%) [7].

Kyzas reported that OMFS literatures lack high-quality evidence articles [8].

Mead and colleagues [9] published a similar literature review in 2005 for clinical studies related to endodontic surgery. They reported that endodontic literature lacks studies at the highest level of evidence and that the vast majority of literature consists of low-level case series.

Since the list of publications presenting the evidence of articles in the field was quite short and therefore insufficient [10], the present study was designed.

The aim of this study was to classify all the articles published in the two highest impact factor journals in the field of endodontics International Endodontic Journal (Int Endod J) and Journal of Endodontics (J Endod) in 2000, 2006 and 2010 according to their level of evidence and the research method.

## 2. Materials and Methods

All the papers published in two leading endodontic journals (J Endod and Int Endod J) in 2000, 2006 and 2010 were included. Articles were read-through and classified into six levels of evidence (LOE). The LOE rating scale used in this article was developed by RDC (Table 1). To simplify the classification, the subgroups of each level were joined and the non-evidence based articles were scored as 0. At last there were 6 groups as 0, 1, 2, 3, 4 and 5.

**Table 1.** Level of evidence (LOE) according to Oxford scale

	Study Type
**1a**	Systematic review (withhomogenicity) of randomized clinical trial(s)
**1b**	Individual randomized clinical trial (with narrow confidence interval)
**2a**	Systematic review with homogenicity of cohort study
**2b**	Individual cohort study
**2c**	"Outcome" research
**3a**	Systematic reviews with homogenicity of case-control studies
**3b**	Individual case-control study
**4**	Case series (and poor quality cohort and case-control studies)
**5**	Expert opinion without explicit critical appraisal

Articles such as technical notes, news, book reviews, animal studies, laboratory studies, tutorials, and letters and case reports were not considered as evidence according to the scale and were therefore classified out of the levels as non-evidence (LOE 0). The authors carried out level categorization and data input and in case of differences in classifications, uncertainties were settled by consensus. Data extraction consisted of the name of the journal, study design, year of publication and level of evidence. Data was analyzed using SPSS 17.0 by Fisher’s exact test to compare LOEs between two journals and among different years.

## 3. Results

**Figure 1.**
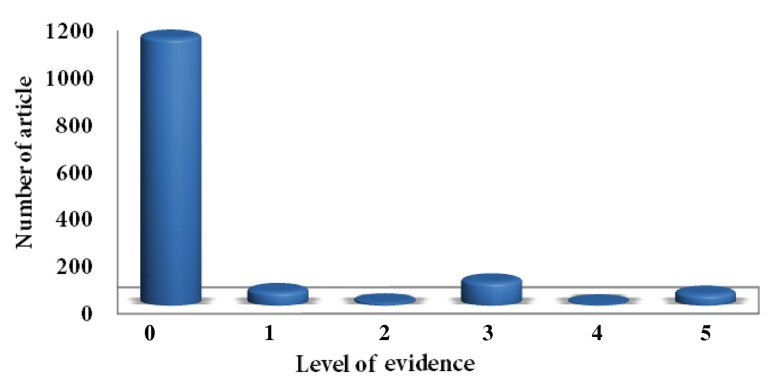
Number of articles in each LOE (0-5)

Two major journals in the field of endodontics were included in the study and a total of 1357 articles were assessed of which 390 were from International Endodontic Journal and 967 from Journal of Endodontics. Of all the studies evaluated, 58 were level 1 (4.3%), 12 (0.9%) were level 2, 99 were level 3 (7.3%), 6 were level 4 (0.4%) and 47 were level 5 (3.5%). Also, 1135 (83.6%) of the articles were classified as non-evidence (Figure 1).

The publication pattern of the articles concerning the study design in different years is shown in Table 2. Among these articles, the percentages of randomized clinical trials and systematic reviews have increased, while the percentage of in-vitro studies has decreased in 2010 compared to 2006 and 2000. The cumulative percentages of RCT and systematic review articles, which carry the highest LOE, in 2010 had marginally significant difference compared to the percentage of articles in 2006 and 2000 (P=0.08).

**Table 2.** Number (n) and percent (%) of various study designs of both journals in different years

Study Design																		
			**In vitro**	**Animal Study**	**RCT**	**Cohort**	**Case Report**	**Case Series**	**Case Control**	**Review**	**Systematic Review**	**Letter**	**Outcome Study**	**Cross Sectional**	**Editorial**	**News**	**Book Review**	**Total**
**year**	**2000**	**n**	143	15	8	0	31	0	0	5	0	0	2	23	2	27	4	260
		**%**	55.0	5.8	3.1	0.0	11.9	0.0	0.0	1.9	0.0	0.0	0.8	8.8	0.8	10.4	1.5	100.0
	**2006**	**n**	218	14	12	9	30	5	3	13	3	6	1	35	2	101	1	453
		**%**	48.1	3.1	2.6	2.0	6.6	1.1	0.7	2.9	0.7	1.3	0.2	7.7	0.4	22.3	0.2	100.0
	**2010**	n	288	47	24	8	43	2	1	16	9	4	2	39	15	140	6	644
		**%**	44.7	7.3	3.7	1.2	6.7	0.3	0.2	2.5	1.4	0.6	0.3	6.1	2.3	21.7	0.9	100.0
**Total**		**n**	649	76	44	17	104	7	4	34	12	10	5	97	19	268	11	1357
		**%**	47.8	5.6	3.2	1.3	7.7	0.5	0.3	2.5	0.9	0.7	0.4	7.1	1.4	19.7	0.8	100.0

Comparison of the LOE of the journals did not reveal statistically significant differences between the published articles (P=0.84).

## 4. Discussion

Evidence based practice has become an essential but great challenge in all fields of medicine. The concept is also being adopted by the dental community [11].

Endodontics has remained a cornerstone in the foundation for dental restorative care in the present century. Unfortunately, there is still substantial shortage of good unbiased studies to be used as an evidence base in clinical endodontics, which is considered a significant problem in endodontics, to which a treatment is given on the basis of opinions and experiences rather than science [11].

University of Detroit Mercy School of Dentistry has developed an evidence-based endodontic literature (EBE) database, which is an attempt to solve the limitation of high-quality sources of information and also to provide the dental community with an educational evidence tool to help justify historical and current endodontic treatment decisions [12]. This endeavor shows the importance of the subject in an era of expanding literature.

In this study most of the articles were in vitro tests (47.8%), while only 3.2% were RCT studies. The majority of the articles (83.6%) were categorized as non-evidence.

It is obvious that different qualities of articles should be expected in different fields of dentistry. For example, in dental materials most of the publications are about the substances and their characteristics and qualities; therefore, most research studies are laboratory tests which do not meet the LOE criteria.

Also animal studies sometimes need to be undertaken before clinical trials. In fields such as endodontics different aspects of the field may be investigated might be discussed and various types of studies required to be planned.

According to Lau and Samman, 50% of articles in oral and maxillofacial surgery journals were non-evidence [6] . In the medical field Proescholdt et al. addressing the benefits of gross total removal of brain tumors concluded that no studies with a high LOE were available [13]. Torabinejad and colleagues [14-16], searched for clinical articles pertaining to the success and failure of surgical and non-surgical endodontic procedures, as well as retreatment and assigned level of evidence to these studies. They confirmed that the majority of the articles evaluated were low-LOE articles.

The high percentage of laboratory and animal projects is attributed to rapid expansion and improvements in endodontic technology and instruments. To explore the safety and unanticipated effects of a new intervention technique, researchers carry out this type of study. While RCT studies provide essential new knowledge for the benefit of the patient, they are usually very costly and time-consuming. In addition, it is generally difficult to persuade patients to comply with the trial of new methods based on the results of animal studies. However, RCT studies should not be the only basis for making decisions about patient care. Results from RCT studies should also be weighed up with the physician’s experience [14, 17].

It was interesting to see a gradual increase in the number of high-LOE articles published in both journals (J. Endod and Int Endod J); however, there was no significant difference between the journals. Overall there was a greater number of articles published in J Endod compared to Int Endod J; agreeing with previous report [18]. Most of the leading journals are interested in publishing more clinical research projects. Researchers are also more aware of the laudable trend greater need of evidence-based dentistry.

It is suggested that future research studies should place great emphasis on systematic reviews and RCT studies compared to non-evidence projects [19]. In areas where there is a genuine of lack of evidence, laboratory and animal studies must be set up. It is probable that RCT studies provide the most appropriate information for patients and basic researches (e.g. in vitro molecular and cell biology experiments, animal studies, material research) and provide essential new knowledge for the benefit of patients as well [12].

## 5. Conclusion

In conclusion, there is an increasing trend in the number of articles with high level of evidence in 2010 compared to previous years. However it seems there deficit of concrete articles that answer clinician’s questions in the endodontic field. Endodontic publications have a long way to go to provide high-LOE articles. Needless to say, journals, authors, editors and endodontists should all cooperate to achieve this goal.

Further researches are warranted as well as focused training of clinical researchers to provide them with enough resources to make significant progress for endodontics and dentistry.
